# Oral high-dose sucrosomial iron vs intravenous iron in sideropenic anemia patients intolerant/refractory to iron sulfate: a multicentric randomized study

**DOI:** 10.1007/s00277-020-04361-3

**Published:** 2020-12-02

**Authors:** Giulio Giordano, Mariasanta Napolitano, Valeria Di Battista, Alessandro Lucchesi

**Affiliations:** 1 Division of Internal Medicine, Hematology Service, Regional Hospital “A. Cardarelli”, Campobasso, Italy; 2 Department of Health Promotion, Mother and Child Care, Internal Medicine and Medical Specialties (PROMISE), Haematology Unit, University Hospital “P. Giaccone”, University of Palermo, Via del Vespro 127, 90127 Palermo, Italy; 3 Hematology Unit, Istituto Scientifico Romagnolo per lo Studio e la Cura dei Tumori (IRST) IRCCS, Meldola, Italy

**Keywords:** Iron deficiency anemia, Intravenous sodium ferrigluconate, Oral Sucrosomial iron, High doses, Refractoriness/intolerance to oral iron sulfate

## Abstract

Iron deficiency anemia is among the most frequent causes of disability. Intravenous iron is the quickest way to correct iron deficiency, bypassing the bottleneck of iron intestinal absorption, the only true mechanism of iron balance regulation in human body. Intravenous iron administration is suggested in patients who are refractory/intolerant to oral iron sulfate. However, the intravenous way of iron administration requires several precautions; as the in-hospital administration requires a resuscitation service, as imposed in Europe by the European Medicine Agency, it is very expensive and negatively affects patient’s perceived quality of life. A new oral iron formulation, Sucrosomial iron, bypassing the normal way of absorption, seems to be cost-effective in correcting iron deficiency anemia at doses higher than those usually effective with other oral iron formulations. In this multicentric randomized study, we analyze the cost-effectiveness of intravenous sodium ferrigluconate vs oral Sucrosomial iron in patients with iron deficiency anemia refractory/intolerant to oral iron sulfate without other interfering factors on iron absorption.

## Introduction

Iron deficiency anemia (IDA) is among the five most frequent causes of disability in humans [1]. Anemia affects one-third of the world population and IDA is the first cause, regarding 1.24 billion of individuals [1, 2]. WHO estimates that 50% of anemia cases worldwide are due to IDA [3] and prevalence of iron deficiency anemia in Europe was estimated about 20% [4, 5].

Iron deficiency (ID) manifests in two forms: absolute or functional. The first one is due to a total body iron stores lack. The second one is present in diseases in which total body iron stores are normal or increased, but iron supply to the bone marrow is inadequate because of reduced iron bioavailability, as in many acute and chronic inflammatory diseases. Absolute and functional iron deficiencies can co-exist [6].

Sucrosomial® iron (SI) is a new oral iron formulation, in which ferric pyrophosphate is protected by a phospholipid bilayer membrane composed mainly by sunflower lecithin, enveloped in a sucrester matrix stabilized by other ingredients as tricalcium phosphate and starch, forming the “sucrosome,” absorbed as a vesicle-like structure, through intestinal M cells, through a paracellular or intracellular way [7].

Sodium ferric gluconate (SFGC) is the cheapest and a safe formulation of intravenous iron currently on the market. Each 5-ml vial contains 177 mg of SFGC, corresponding to 62.5 mg of trivalent elemental iron.

The aim of the current study was to verify whether high doses of SI are as safe, effective, cheap, and well tolerated as the standard SFGC intravenous doses in patients with iron deficiency anemia, without significant comorbidities.

## Patients and methods

This study is a prospective multicentric study. Patients were enrolled from 3 centers: two regional hospitals and one regional family medicine service. Time of enrollment was 20 months.

Demographic characteristics of the enrolled patients are listed in Table 1. The median follow-up was 12 months (range, *R*: 8–18).

**Table 1:** Patients’ characteristics

	Group A	Group B	*p*
*N*	45	45	
M/F	2/3	2/3	n.s.
Median age (years)	75 (*R*: 35–85)	62 (*R*: 40–83)	0.02
Age > 80 years old	14	5	0.04
Gastric bleeding	15	18	n.s.
Intestinal bleeding	8	6	n.s.
Menometrorrhagia	22	21	n.s.
Median Hb (g/dl)	8.5 (*R*: 6.5–10)	8.2 (*R*: 7.5–9.5)	n.s.
Ferritin (ng/ml)	5 (*R*: 3–21)	7 (*R*: 2–19)	n.s.
Iron sulfate intolerance	18	7	0.02
Iron sulfate ineffectiveness	22	30	n.s.
Median days of dark stools	5 (*R*: 2–12)	6 (*R*: 1–8)	n.s.
Pat. with > 7 days of dark stools	5	2	n.s.
No. of patients taking PPI	20	21	n.s.
Median dose of elemental iron received (mg)	3360 (*R*: 2800–3600)	640 (*R*: 520–780)	< 0.01

Patients were consecutively randomized (random 1:1) to receive oral Sucrosomial iron (SI) or i.v. sodium ferric gluconate.

In group A, patients received Sucrosomial iron (Sideral® Forte) 30-mg tablet 2 tabs twice daily for 1 month; for the first 15 consecutive days, iron was taken without food; and for the following 15 days with food, with or without antiacid therapy.

In group B, patients received sodium ferric gluconate 1 vial (62.5 mg) in normal saline solution (ns) 250 ml intravenous (i.v.) under continuous infusion (ci) in 3 h/day. The number of required vials was calculated with the adapted formula: (0.65 × body weight in kg) (12-Hb g/dl) × 3.3 × 1.5 / 62.5 [8, 9].

The study was carried out in accordance with the provisions of the Declaration of Helsinki and local regulations. Informed consent was obtained from each participant. The protocol was approved by the institutional review boards of all the enrolling centers.

Inclusion criteria were age > 18 years, no other established causes of anemia, hemoglobin < 10 g/dl, ferritin < 30 ng/ml, total iron binding capacity saturation < 20%, iron sulfate intolerance, failure to increase 1 g of hemoglobin (Hb) after 1-month treatment with iron sulfate at a dosage of 660 mg (2 tabs)/day, failure of normalization of the Hb level after 3 months of 660 mg of iron sulfate (2 tabs), at least one positive sample of fecal occult blood test out of three with esophageal gastro-duodenoscopy and pan colonoscopy negative for neoplasia, non-neoplastic hypermenorrhea, and/or menometrorrhagia, gastric bleeding (ulcer, gastritis, angiodysplasia), small intestine bleeding (angiodysplasia, ulcer), and colorectal bleeding (diverticulitis, angiodysplasia, hemorrhoids).

Exclusion criteria were loss of 1 g Hb/dl within a timeframe of less than 7 days, hyperthyroidism, severe chronic heart failure, autoimmune disease, inflammatory bowel disease, ischemic/hemorrhagic enterocolitis, neoplasia with or without active treatment, helminthic infestation, erythrocyte sedimentation rate (ESR) and/or C-reactive protein (CRP) increase, pregnancy/breastfeeding, and severe organ failure.

The median cost of treatment in each group was calculated taking into account the overall cost of all therapy for each patient during the observation period, then dividing this cost for the months of follow-up to obtain average cost per month of follow-up per patient. In each group, the median of the average cost per month of follow-up per patient was then calculated. This provides a valuation of the costs, unrelated to the exact cost of the drug, but linked to the outcome and efficacy of the therapeutic strategy during the observation period.

Details of the calculated costs are reported in Table 2.

**Table 2:** Calculated costs of iron treatment

CBC + blood tests + iron balance+ B_12_ + folates + disposable materials (needle + gauzes + test tube + disinfectant) + ticket: 70 €	
Effluent: 0.43 €	
Normal saline 250 cc: 0.90 €	
Gauzes: 0.01 €	
1 day of day hospital stay (including nursing care and meals): 200 €	
Medical care for 1 day of day hospital: 140 €	
1 working day lost: 584 €	
Ticket for medical outpatient visit: 25 €	
Fee for outpatient examining room: 16 €/die	
Cost 1 U PRBC or PLT: approximately 200 €	
Cost 1 tablet Sucrosomial iron: 1 €	
Cost 1 sodium ferrigluconate vial: 1.2 €	

Evaluation of perceived quality of life was performed by administering the SF-8™ questionnaire, a generic multipurpose short-form health-related quality of life instrument, developed by the RAND Corporation and the Medical Outcomes Study (MOS).

A numerical rating scale (NRS) between 0 and 10 was used to assess pain intensity (0: no pain, 10: the worst pain ever possible) [10].

The degree of diarrhea was classified according to the NCI Common Toxicity Criteria (CTC) scale, version 3.0.

The following assays were weekly performed: complete blood count, B_12_, folates, haptoglobin, blood iron, ferritin, total iron binding capacity, liver enzymes, creatinine, blood urea nitrogen, C-reactive protein in the first 6 weeks of the study. All these tests were performed monthly during the entire follow-up period with fecal occult blood test.

We analyzed differences between clinical parameters among 90 patients to evidence any possible association and significant difference. Depending on distributions, variables were described as mean and median. To compare distributions between groups, we used one-way ANOVA and post hoc comparisons were performed with the unpaired-samples *t* test, Mann-Whitney test, or chi-square test, as appropriate; in all cases, we used a Bonferroni correction. The chi-square test was computed with Yates’ continuity correction for 2 × 2 contingency tables. A *p* value < 0.05 was considered statistically significant.

## Results

**Fig. 1:**
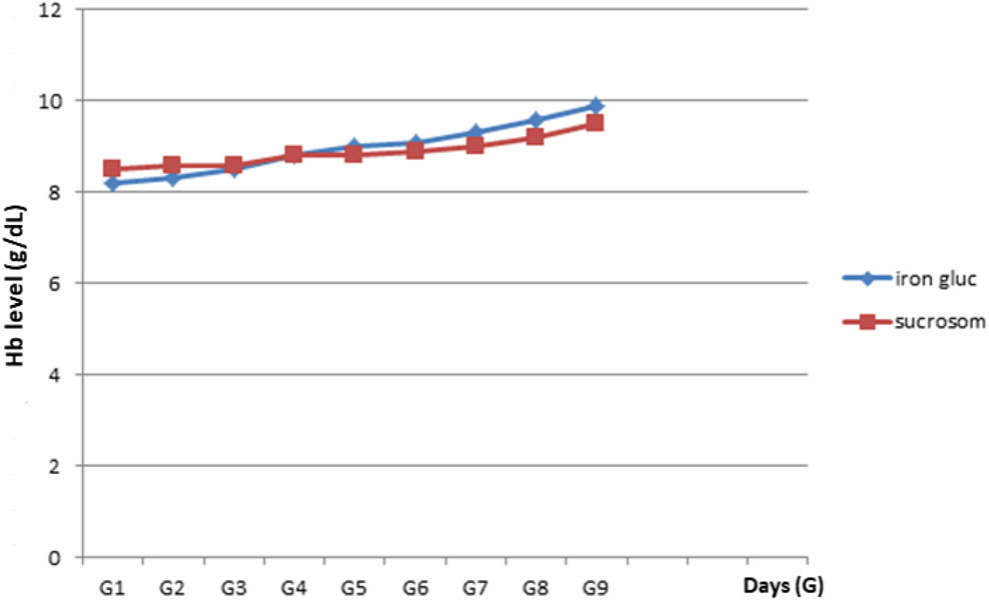
Days needed to obtain Hb 1-g/dl increase from basal level. iron gluc, sodium ferrigluconate; sucrosom, Sucrosomial iron

The median number of sodium ferric gluconate vials administered was 10 (*R*: 8–12). Days needed to reach a 1-g/dl increase of hemoglobin levels from basal level were 9 days (*R*: 7–15) in group A (Sucrosomial iron) vs 7 days (*R*: 6–11) in group B (SFGC) (*p* n.s., Fig. 1).

**Fig. 2:**
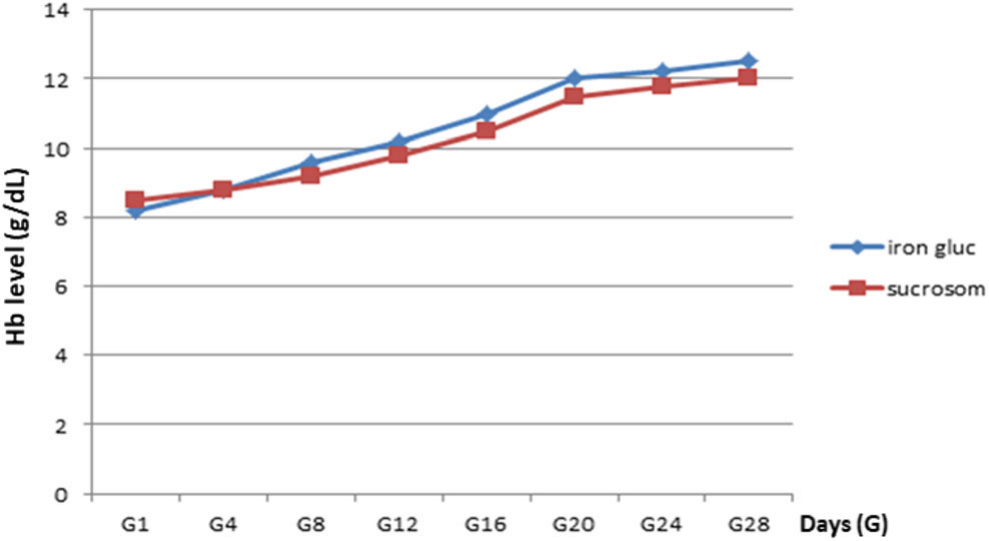
Days needed to achieve Hb 12-g/dl target level. iron gluc, sodium ferrigluconate; sucrosom, Sucrosomial iron

Weeks required to achieve an Hb target value of 12 g/dl were 4 (*R*: 2–4) in group A vs 3.5 (*R*: 1.5–4) in group B (*p* n.s., Fig. 2).

In the group of patients taking oral Sucrosomial iron without food, 7 (16%) complained of stomach pain (NRS evaluation: 2 patients = 4, 2 patients = 3, and 3 patients = 2) and 5 (11%) diarrhea (3 patients = G2, 2 patients = G1) (Table 3). All patients complaining side effects were 80 years old or more. When patients took Sucrosomial iron with food, 2 (5%) complained burning stomach pain (NRS evaluation = 2) and 2 (5%) diarrhea (NCI scale = G1).

**Table 3:** Reported side effects following iron administration

	Sucrosomial iron w/o food	Sucrosomial iron with food	SFGC
No. of patients with side effects/total	12/45	4/45	10/45
Patients with side effects ≥ 80 years old/total	12/45	4/45	2/45
Stomach pain	7	2	0
Diarrhea	5	2	0
Hypotension	0	0	5
Headache	0	0	5

In the SFGC group, 5 (11%) patients showed hypotension and 5 urticaria and headache. Only 2 patients complaining side effects were older than 80 years.

The highest total monthly median expenditures, accounting for 300 Euros, were reported in patients treated with SFGC, compared to patients supplemented with SI (*p* < 0.001), accounting for 120 Euros. Although the unitary cost of a Sideral® pill box seems to be higher than that of a vial of Ferlixit® (EUR 20 per Sideral® box vs EUR 0.95/vial for i.v. iron), the highest direct (EUR 35 for doctor visit, EUR 35 for daily hospital admission, EUR 10 for outpatient hospital visit) and indirect (EUR 120 for a working day loss) costs were seen in the SFGC group, with a significant statistical difference (*p* = 0.013).

At SF-8 evaluation, patients receiving SI showed a score of 58 vs 42 in the SFGC group; thus, a better quality of life and wellness perception was recorded in the SI group.

At a median time of 12 months after enrollment, the median value of ferritin (ng/ml) at end of treatment was 260 (*R*: 150–320) in the SI group vs 18 (*R*: 5–56) in the SFGC group (*p* = 0.01).

Seventeen (37%) patients in the SI group and 16 (35%) in the SFGC group (*p* n.s.) showed at least 3 fecal occult blood tests positive during the follow-up period, after the first 6 weeks of study.

Fifteen (33%) patients in the SI group and 10 (22%) in the SFGC group (*p* n.s.) showed at least 3 increased values of C-reactive protein with respect to the normal range during the follow-up period, after the first 6 weeks of study.

## Discussion

IDA is a public health problem affecting 32.8% of premenopausal and 40.1% of pregnant women, 41.7% of growing children [1], between 13 and 90% of patients with various medical diseases [11–18], and 30% of elderly people [19]; furthermore, approximately two-thirds of patients undergoing major surgery develop postoperative anemia [20–22].

The real limit of oral iron supplements is their absorption. In fact, it happens like through a funnel. The heme-iron is absorbed by a heme transporter [23], ferric iron is absorbed by the DMT1 (divalent metal transport 1) transporter [24–28], lactoferrin binds iron through a lactoferrin-binding protein/receptor [29–31], and ferritin iron can be taken up via an endocytic process in Caco-2 cells (which have some enterocyte-like characteristics) and probably also in vivo [32–34]. All these iron carriers can be heavily upregulated, as DMT1, up to 4–12-fold during iron deficiency anemia and erythropoietin increase [35, 36]. However, these receptors, also when upregulated, allow a limited iron absorption. The iron efflux from enterocytes is regulated by ferroportin that likely allows a maximum increase in iron absorption of 3–4 folds, as seen in thalassemic patients [37, 38]. Moreover, in iron-deficient patients, the administration of oral iron sulfate causes an increase of serum hepcidin, a hormone blocking ferroportin and protecting against iron overload, that falls to basal levels 48 h after treatment, thus imposing an every-other-day administration schedule for an optimal iron absorption [39].

A typical Western diet provides about 15 mg of iron per day and only 10% of it is absorbed [36]. Recovery from blood loss causes an increase in iron absorption in the duodenum up to 20-fold. Patients with severe IDA or with normal iron stores receiving erythropoietin can absorb up to 20 to 40 mg/day of iron and the amount decreases when Hb levels increase [40, 41].

Oral iron is a cheap and effective way of correcting the martial deficiencies in patients without comorbidities, when iron loss exceeds the iron amount absorbed through food in the small intestine.

The cheapest commercially available oral iron formulation in Italy is iron sulfate. It is absorbed via a DMT1 transporter localized in the duodenal enterocyte brush border. Each tablet contains 325 mg of iron salts, containing 65 mg of elemental iron [35, 42]. However, gastrointestinal side effects are common [9, 43, 44]. It is estimated that 30% or more of patients taking iron-containing oral preparations develop gastrointestinal symptoms like nausea, constipation, diarrhea, dark stools, epigastric pain, and vomiting [45–47]. Gastrointestinal symptoms are proportional to the amount of elemental iron ingested [48].

Sucrosomial® iron is a new oral iron formulation, in which ferric pyrophosphate is protected by a phospholipid bilayer membrane composed mainly by sunflower lecithin, enveloped in a sucrester matrix stabilized by other ingredients as tricalcium phosphate and starch, forming the “sucrosome,” absorbed as a vesicle-like structure, through intestinal M cells, through a paracellular or intracellular way, bypassing the conventional iron DMT-1 absorption pathway [7, 49, 50]. Each tablet contains 30 mg of pyrophosphate iron.

The use of intravenous (i.v.) iron is indicated in patients intolerant to oral iron or when there is a problem of iron absorption (as in celiac disease, inflammatory bowel disease, gastric bypass, chronic inflammatory disease, cancer chemotherapy) or when intestinal blood losses exceed the ability of the normal duodenum to absorb ingested iron (as in obstetrics or surgical blood loss or in Osler-Weber-Rendu syndrome), because it bypasses mechanisms of duodenal absorption and increases Hb levels more quickly than oral iron [51–55].

In the current study, it is noteworthy that the number of days required to achieve a 1-g increase in hemoglobin levels after enrollment and the time to reach target hemoglobin level of 12 g/dl is practically identical in the two treatment groups. It seems that for the SI group, the iron given in the Sucrosomial formulation bypassed all the usual absorption mechanisms present in the brush border membrane of enterocytes, behaving, therefore, almost like the SFGC delivered parenterally.

This result is in open contrast to what would be expected by administering any other oral iron formulation. For example, we can consider the case of oral iron sulfate. The median amount of iron needed to achieve an increase of 1 g/dl of hemoglobin based on the aforementioned formula would be 225 mg. Considering that each iron sulfate tablet contains 65 mg of elemental iron, of which a maximum of 25 mg is absorbed, and that the optimal administration schedule is 1 tablet every other day, 18 days of treatment would be needed theoretically to achieve the desired goal vs 9 days in the SI group and 7 days in the SFGC group. Furthermore, to achieve the target Hb level of 12 g/dl in 72 days of treatment with iron sulfate should be needed vs 28 days in the SI group and 24 days in the SFGC group.

Some doubts can arise from the high amount of elemental iron received in the SI group in a month; in fact, it could potentially cause hemosiderosis. However, if we consider that the quantity of elemental iron necessary to reach the median level of 12 g/dl of hemoglobin, reached in 1 month in the SI group, is a median of 900 mg, that the median continuous loss of hemoglobin in each patient was 2 g/dl per month, corresponding to a median of 450 mg of elemental iron, and that 1000 mg of iron is needed to replenish martial stocks, the total median iron needed is 2350 mg. Moreover, in feces, humans loss about 13% of lipid and sugar ingested [56], corresponding to 18 tablets of Sideral loss in stools each month, for a total of 540 mg of elemental iron; thus, the total median requirement for each patient in the SI group monthly is about 2800 mg. This might explain why at a median time of 12 months from enrollment the median value of ferritin at end of treatment was 260 ng/ml (*R*: 150–320) in the SI group, without any evidence of hemosiderosis.

Results in patients taking proton pump inhibitors (PPIs) were superimposable, without statistically significant differences, to those not taking PPIs in each treatment group. This is in contrast with the evidence that proton pump inhibitors affect iron absorption, at least for the SI group [57–61].

Gastrointestinal side effects, mainly of low level, were reported in 27% of patients from the SI group, in accordance with data already reported in the literature [47]. It is not surprising that the totality of patients with side effects are 80 years old or older. In fact, 40% of elderly patients show gastrointestinal side effects [48, 62] and probably the high number of octogenarians with gastrointestinal side effects is only due to the low number of patients in our study. On the other hand, it is noteworthy that in patients taking Sucrosomial iron with food, gastrointestinal side effects are reduced to almost one-third (only 10%), a markedly lower percentage in comparison to previous studies.

Equally important is the fact that the administration of Sucrosomial iron with food does not impair its absorption, as shown by the same effectiveness of intravenous iron. In fact, usually, when oral iron formulation is given with food, its absorption is impaired by calcium, polyphenols, phytates, eggs, or soy contained in aliments [63].

ID, in which IDA is included [64], causes physical productivity reduction and cognitive and physical disability with an economic loss quantified, in 2002, as an absolute dollar value of $4.2 billion annually (corresponding to € 3.8 billion), with reference only to physical productivity losses in South Asia [64]. Economic loss secondary to ID has been estimated, in 2019, as CHF 78 million (€ 73.12 million) for direct medical costs and between CHF 26 million (€ 24.37 million) and CHF 33 million (€ 31 million) in a Swiss female population [65, 66].

The economic evaluation of SI treatment–related costs in the current study showed a possible cost saving, translated into a 60% abatement of overall monthly expenditures per patient compared to the SFGC group. Indeed, although the treatment cycle with Sucrosomial iron had higher purchase costs compared to i.v. iron, it greatly reduced other direct clinical expenditures (cost minimization) for the hospital and the public healthcare system as well as indirect costs. The information acquired by comparing the costs of the two different therapies confirms what our group has already observed in the context of myelodysplastic syndromes. In that case too, the high number of hospital admissions resulted in an increase in indirect costs and losses deriving from patients’ absences from work [67].

Moreover, a warning issued by the EMA and by the Italian Drugs Agency (AIFA) advises against the use of i.v. iron outside hospital with an intensive care unit, given the potentially fatal side effects linked to i.v. hypersensitization [68]. This imposes SFGC administration only in hospital, considerably worsening quality of life of patients receiving SFGC and explaining the higher value of perceived quality of life in the SI group.

## Data Availability

All data related to this study are available on request.

## References

[1] GBD 2016 (2017). Disease and Injury Incidence and Prevalence Collaborators. Global, regional, and national incidence, prevalence, and years lived with disability for 328 diseases and injuries for 195 countries, 1990-2016: a systematic analysis for the Global Burden of Disease Study 2016. Lancet.

[2] Kassebaum NJ, Jasrasaria R, Naghavi M, Wulf SK, Johns N, Lozano R, Regan M, Weatherall D, Chou DP, Eisele TP, Flaxman SR, Pullan RL, Brooker SJ, Murray CJL (2014). A systematic analysis of global anemia burden from 1990 to 2010. Blood.

[3] WHO (2001) Iron deficiency anemia assessment, prevention, and control: a guide for programme managers. Geneva: World Health Organization WHO/NHD/01.3

[4] Terrier B, Resche-Rigon M, Andres E, Bonnet F, Hachulla E, Marie I, Rosenthal E, Cacoub P, Groupe de Recherche sur les Anémies en Médecine Interne (2012). Prevalence, characteristics and prognostic significance of anemia in daily practice. QJM.

[5] Eisele L, Dürig J, Broecker-Preuss M (2013). Prevalence and incidence of anemia in the German Heinz Nixdorf Recall Study. Ann Hematol.

[6] Wish JB (2006). Assessing iron status: beyond serum ferritin and transferrin saturation. Clin J Am Soc Nephrol Suppl.

[7] Fabiano A, Brilli E, Fogli S (2018). Sucrosomial® iron absorption studied by in vitro and ex-vivo models. Eur J Pharm Sci.

[8] Price E, Artz AS, Barnhart H (2014). A prospective randomized wait list control trial of intravenous iron sucrose in older adults with unexplained anemia and serum ferritin 20-200 ng/mL. Blood Cells Mol Dis.

[9] Alleyne M, Horne MK, Miller JL (2008). Individualized treatment for iron-deficiency anemia in adults. Am J Med.

[10] Glossary (2000). Spine.

[11] Jankowska EA, Rozentryt P, Witkowska A (2010). Iron deficiency: an ominous sign in patients with systolic chronic heart failure. Eur Heart J.

[12] Cohen-Solal A, Damy T, Terbah M (2014). High prevalence of iron deficiency in patients with acute decompensated heart failure. Eur J Heart Fail.

[13] Klip IT, Comin-Colet J, Voors AA (2013). Iron deficiency in chronic heart failure: an international pooled analysis. Am Heart J.

[14] Okonko DO, Mandal AK, Missouris CG, Poole-Wilson PA (2011). Disordered iron homeostasis in chronic heart failure: prevalence, predictors, and relation to anemia, exercise capacity, and survival. J Am Coll Cardiol.

[15] Yeo TJ, Yeo PS, Ching-Chiew Wong R (2014). Iron deficiency in a multi-ethnic Asian population with and without heart failure: prevalence, clinical correlates, functional significance and prognosis. Eur J Heart Fail.

[16] Peyrin-Biroulet L, Williet N, Cacoub P (2015). Guidelines on the diagnosis and treatment of iron deficiency across indications: a systematic review. Am J Clin Nutr.

[17] McClellan W, Aronoff SL, Bolton WK (2004). The prevalence of anemia in patients with chronic kidney disease. Curr Med Res Opin.

[18] Ludwig H, Muldur E, Endler G, Hubl W (2013). Prevalence of iron deficiency across different tumors and its association with poor performance status, disease status and anemia. Ann Oncol.

[19] Stauder R, Valent P, Theurl I (2018). Anemia at older age: etiologies, clinical implications, and management. Blood.

[20] Munoz M, Laso-Morales MJ, Gomez-Ramirez S, Cadellas M, Nunez-Matas MJ, Garcia-Erce JA (2017). Pre-operative haemoglobin levels and iron status in a large multicentre cohort of patients undergoing major elective surgery. Anaesthesia.

[21] Munoz M, Acheson AG, Auerbach M (2017). International consensus statement on the peri-operative management of anaemia and iron deficiency. Anaesthesia.

[22] Musallam KM, Tamim HM, Richards T (2011). Preoperative anaemia and postoperative outcomes in non-cardiac surgery: a retrospective cohort study. Lancet.

[23] Shayeghi M, Latunde-Dada GO, Oakhill JS (2005). Identification of an intestinal heme transporter. Cell.

[24] Fleming MD, Trenor CC, Su MA (1997). Microcytic anaemia mice have a mutation in Nramp2, a candidate iron transporter gene. Nat Genet.

[25] Gunshin H, Mackenzie B, Berger UV (1997). Cloning and characterization of a mammalian proton-coupled metal-ion transporter. Nature.

[26] Mackenzie B, Garrick MD (2005). Iron imports. II. Iron uptake at the apical membrane in the intestine. Am J Physiol Gastrointest Liver Physiol.

[27] Gulec S, Anderson GJ, Collins JF (2014). Mechanistic and regulatory aspects of intestinal iron absorption. Am J Physiol Gastrointest Liver Physiol.

[28] Gulec S, Collins JF (2014). Molecular mediators governing iron-copper interactions. Annu Rev Nutr.

[29] Iyer S, Lonnerdal B (1993). Lactoferrin, lactoferrin receptors and iron metabolism. Eur J Clin Nutr.

[30] Suzuki YA, Shin K, Lonnerdal B (2001). Molecular cloning and functional expression of a human intestinal lactoferrin receptor. Biochemistry.

[31] Shin K, Wakabayashi H, Yamauchi K, Yaeshima T, Iwatsuki K (2008). Recombinant human intelectin binds bovine lactoferrin and its peptides. Biol Pharm Bull.

[32] San Martin CD, Garri C, Pizarro F, Walter T, Theil EC, Núñez MT (2008). Caco-2 intestinal epithelial cells absorb soybean ferritin by mu2 (AP2)-dependent endocytosis. J Nutr.

[33] Kalgaonkar S, Lonnerdal B (2009). Receptor-mediated uptake of ferritin- bound iron by human intestinal Caco-2 cells. J Nutr Biochem.

[34] Theil EC, Chen H, Miranda C (2012). Absorption of iron from ferritin is independent of heme iron and ferrous salts in women and rat intestinal segments. J Nutr.

[35] Zimmermann MB, Biebinger R, Egli I, Zeder C, Hurrell RF (2011). Iron deficiency up-regulates iron absorption from ferrous sulphate but not ferric pyrophosphate and consequently food fortification with ferrous sulphate has relatively greater efficacy in iron-deficient individuals. Br J Nutr.

[36] Finch C (1994). Regulators of iron balance in humans. Blood.

[37] Fiorelli G, Fargion S, Piperno A, Battafarano N, Cappellini MD (1990). Iron metabolism in thalassemia intermedia. Haematologica.

[38] Gardenghi S, Marongiu MF, Ramos P (2007). Ineffective erythropoiesis in beta-thalassemia is characterized by increased iron absorption mediated by down-regulation of hepcidin and up-regulation of ferroportin. Blood.

[39] Stoffel NU, Zeder C, Brittenham GM, Moretti D, Zimmermann MB (2020). Iron absorption from supplements is greater with alternate day than with consecutive day dosing in iron-deficient anemic women. Haematologica.

[40] Norrby A (1974). Iron absorption studies in iron deficiency. Scand J Haematol Suppl.

[41] Skikne BS, Cook JD (1992). Effect of enhanced erythropoiesis on iron absorption. J Lab Clin Med.

[42] Boggs DR (1987). Fate of a ferrous sulfate prescription. Am J Med.

[43] Crosby W (1984). The rationale for treating iron deficiency anemia. Arch Intern Med.

[44] DeLoughery T (2014). Microcytic anemia. N Engl J Med.

[45] Kruske SG, Ruben AR, Brewster DR (1999). An iron treatment trial in an aboriginal community: improving non-adherence. J Paediatr Child Health.

[46] Tolkien Z, Stecher L, Mander AP, Pereira DI, Powell JJ (2015). Ferrous sulfate supplementation causes significant gastrointestinal side-effects in adults: a systematic review and meta-analysis. PLoS One.

[47] Cancelo-Hidalgo MJ, Castelo-Branco C, Palacios S (2013). Tolerability of different oral iron supplements: a systematic review. Curr Med Res Opin.

[48] Rimon E, Kagansky N, Kagansky M (2005). Are we giving too much iron? Low-dose iron therapy is effective in octogenarians. Am J Med.

[49] Fievez V, Plapied L, Plaideau C (2010). In vitro identification of targeting ligands of human M cells by phage display. Int J Pharm.

[50] Mabbott NA, Donaldson DS, Ohno H, Williams IR, Mahajan A (2013). Microfold (M) cells: important immunosurveillance posts in the intestinal epithelium. Mucosal Immunol.

[51] Macdougall IC, Bock AH, Carrera F (2014). FIND-CKD: a randomized trial of intravenous ferric carboxymaltose versus oral iron in patients with chronic kidney disease and iron deficiency anaemia. Nephrol Dial Transplant.

[52] Onken JE, Bregman DB, Harrington RA (2014). A multicenter, randomized, active-controlled study to investigate the efficacy and safety of intravenous ferric carboxymaltose in patients with iron deficiency anemia. Transfusion.

[53] Vadhan-Raj S, Strauss W, Ford D (2014). Efficacy and safety of IV ferumoxytol for adults with iron deficiency anemia previously unresponsive to or unable to tolerate oral iron. Am J Hematol.

[54] Auerbach M, Ballard H, Glaspy J (2007). Clinical update: intravenous iron for anaemia. Lancet.

[55] Auerbach M, Goodnough LT, Picard D, Maniatis A (2008). The role of intravenous iron in anemia management and transfusion avoidance. Transfusion..

[56] Guyton and Hall Textbook of medical physiology (2011) Digestion and absorption in the gastrointestinal tract, Saunders 12th Ed, Chap. 65

[57] Tran-Duy A, Connell NJ, Vannmolkot FH (2019). Use of proton pump inhibitors and risk of iron deficiency: a population-based case–control study. J Intern Med.

[58] Lam JR, Schneider JL, Quesenberry CP, Corley DA (2017). Proton pump inhibitor or H-2 receptor antagonist use and iron deficiency. Gastroenterology.

[59] Sarzynski E, Puttarajappa C, Xie Y, Grover M, Laird-Flick H (2011). Association between proton pump inhibitor use and anemia; a retrospective study. Dig Dis Sci.

[60] Amjera AV, Shastri GS, Gajera MJ, Judge TA (2012). Suboptimal response to ferrous sulphate in iron-deficient patients taking omeprazole. Am J Ther.

[61] Hashimoto R, Matsuda T, Chonan A (2014). Iron-deficiency anemia caused by a proton pump inhibitor. Intern Med.

[62] Shah R, Agarwal AK (2013). Anemia associated with chronic heart failure: current concepts. Clin Interv Aging.

[63] Hallberg L, Hulthén L (2000). Prediction of dietary iron absorption: an algorithm for calculating absorption and bioavailability of dietary iron. Am J Clin Nutr.

[64] Napolitano M, Dolce A, Celenza G (2014). Iron-dependent erythropoiesis in women with excessive menstrual blood losses and women with normal menses. Ann Hematol.

[65] Horton S, Ross J (2003). The economics of iron deficiency. Food Policy.

[66] Blank PR, Tomonaga Y, Szucs TD, Schwenkglenks M (2019). Economic burden of symptomatic iron deficiency - a survey among Swiss women. BMC Womens Health.

[67] Giordano G, Cutuli MA, Lucchesi A (2020). Iron support in erythropoietin treatment in myelodysplastic syndrome patients affected by low-risk refractory anaemia: real-life evidence from an Italian setting. Acta Haematol.

[68] European Medicines Agency. New recommendations to manage risk of allergic reactions with intravenous iron-containing medicines. European Medicines Agency, London. Available from: http://www.ema.europa.eu/ema/index.jsp?curl=pages/news_and_events/news/2013/06/newsdetail_001833.jsp&mid=WC0b01ac058004d5c1. Accessed 10 Oct 2020

